# A clinical study of the effect of calcium sodium phosphosilicate on dentin hypersensitivity

**DOI:** 10.4317/jced.50955

**Published:** 2013-02-01

**Authors:** Anirudh B. Acharya, Sai M. Surve, Srinath L. Thakur

**Affiliations:** 1 MDS, Professor, Department of Periodontics, S.D.M. College of Dental Sciences & Hospital, Karnataka, India; 2 MDS, Consultant Periodontist, Private Practice, Mumbai-400103, Borivali, Maharashtra, India; 3 MDS, Professor, Department of Periodontics & Principal of S.D.M. College of Dental Sciences & Hospital, Karnataka, India

## Abstract

Objective: Dentinal hypersensitivity is a commonly encountered problem with varied treatment options for its management. A large number of home use products have been tested and used for the management of dentinal hypersensitivity. This 8 week clinical trial investigates the temporal efficacy of commercially available calcium sodium phosphosilicate containing toothpaste in comparison to a potassium nitrate containing toothpaste. Methods: A total 20 subjects between the ages of 18 to 65 years were screened for a visual analogue score (VAS) for sensitivity of 5 or more by testing with a cold stimulus and randomly divided into test and positive control groups. Baseline sensitivity VAS scores to air evaporative stimulus were recorded for minimum two teeth. The subjects were prescribed respective dentifrices and revaluated for sensitivity scores at 2, 4 and 8 weeks. Results:The study demonstrated reduction in symptoms for all treatment groups from baseline to 2, 4 and 8 weeks. The calcium sodium phosphosilicate group showed a higher degree of effectiveness at reducing hypersensitivity to air evaporative stimulus at 2 weeks, than commercially available potassium nitrate. However, there was no significant difference in scores of subjects using the calcium sodium phosphosilicate toothpaste as compared to potassium nitrate at 4 weeks and 8 weeks. Conclusion: Calcium sodium phosphosilicate showed greater reduction in sensitivity compared to potassium nitrate at an earlier stage which is of high clinical value. However, based on the findings of the present study long term effects of calcium sodium phosphosilicate seem to be less promising than previously claimed.

** Key words:**Dentinal desensitizing agents, dentinal hypersensitivity, toothpaste, pain measurement.

## Introduction

Dentine hypersensitivity (DH) is the term used to describe common, painful condition of the permanent teeth; the etiology of which, however, is still poorly understood and the various mechanisms have been proposed to explain the development of dentinal hypersensitivity and treatment alternatives have been reviewed (1-2). Most accepted of these is the hydrodynamic theory which was first explained by Gysi in 1900 and the experimental evidence for which was provided by Bränström. According to this theory, the movement of dentinal fluid on stimulation with thermal, chemical, evaporative or electric stimulus is responsible for excitation of the underlying dentinal mechanoreceptor resulting in sensitivity. Thus many treatment options aim at achieving dentinal tubule occlusion to prevent dentinal fluid movement, thereby reducing hypersensitivity.

The difficulty found in treating DH is expressed by the enormous number of techniques and therapeutic alternatives to relieve it (3). Several methods and materials have been tried to reduce dental sensitivity, ranging from home-use, over the counter products such as desensitizing mouthwashes, dentifrices or tray application foams to in-office application products such as varnishes, liners, restorative materials, dentinal adhesives iontophoresis procedures and more recently, lasers.

- Home use products- dentifrices

The home use products are most realistic and practical means of treating most patients with mild to moderate dentine hypersensitivity and they generally form the first step in routine management. Among these, desensitizing dentifrices have established themselves as principal home care therapeutic agents owing to the fact that they are readily and widely available, cost effective, simple to use and non-invasive and the habit of tooth brushing being almost universal (4). A large number of agents have been shown to reduce the tubule diameter by precipitation of crystals and also shown to be clinically efficacious.

- Potassium nitrate

Potassium nitrate containing dentifrices have consistently shown benefit in alleviating the hypersensitivity symptoms (5-7). They mainly act by blocking the neural transmission. A review by Kanapka concluded that potassium nitrate use reduces hypersensitivity in 8-12weeks (8).

However as Porto et al. (3) have pointed out that in spite of a large amount of literature it is not possible to reach a consensus about a product that represents the gold standard in the treatment of dentinal hypersensitivity. Nevertheless, a large number of commercially available dentifrices consist of potassium nitrate.

- NovaMin technology

NovaMin was developed in the 1990s as a modification of bioactive glass which then had proven to be useful in bone regeneration and repair. It was found that NovaMin also reacted with the tooth dentin and was modified by grinding the particles, to obtain particles that were small enough to access the dentinal tubules. Each microscopic NovaMin particle serves as a delivery system for these ionized bioactive particles.

When the particle is exposed to fluid: saliva or tap water, it instantly reacts, releasing mineral ions of calcium (Ca) and phosphate (PO4) that augment the natural remineralization process. As the particle reactions continue and the deposition of Ca and PO4 complexes continue, this layer crystallizes into hydroxycarbonate apatite which is chemically and structurally equivalent to biological apatite. The combination of the residual NovaMin particles and the newly formed hydroxycarbonate apatite layer results in the physical occlusion of dentinal tubules, which will relieve hypersensitivity. In-vitro analyses have shown significant occlusion of tubules with the NovaMin compounds (9,10).

Each NovaMin bioglass particle is made of Calcium sodium phosphosilicate with 25% sodium, 25% calcium, 6-8% phosphate and remainder, silica.

Previous studies (10-12) have shown clear benefit of NovaMin containing dentifrice over controls. Also a recent review (13) concluded that the data look promising, but more research is needed.

The present study intends to compare the temporal efficacy of a calcium sodium phosphosilicate containing toothpaste (Vantej® Dr Reddys’s Labs, Hyderabad, India) with potassium nitrate (Sensodent-K®, Warren Phar-maceuticals, Mumbai, India) containing tooth-paste both of which are commercially available.

## Material and Methods

This study was a single center, randomized, double blind and parallel group clinical trial. It was conducted in Department of Periodontics at this institution in accordance with the Declaration of Helsinki and Guidelines for Good Clinical Practice.

The study duration was 8 weeks, in which sensitivity scores were measured at baseline, at 2 weeks, 4 weeks and at 8 weeks. After ethical approval, subjects were selected from the outpatient section of the Department of Periodontics. Duration of the study was from October 2009 to August 2010.

- Inclusion Criteria:

1. Patients need to have at least two sensitive permanent tooth surfaces (buccal/facial aspects of incisors, canines or premolars).

2. Sensitive tooth surfaces are selected if they have wasting diseases and/or gingival recession.

3. No history of periodontal therapy in the past one year.

- Exclusion Criteria:

1. Presently on desensitizing treatment.

2. Subjects with orthodontic appliances or bridge work that may interfere with evaluation.

3. Medical (including psychiatric and pharmacotherapeutic) histories that may compromise study protocol.

4. Allergies.

5. Systemic conditions which are etiologic/predisposing to dentinal hypersensitivity.

6. Eating disorders.

7. Pregnancy or breast feeding.

8. Any dental treatment which may have an effect on the desensitizing agent being used.

9. Any other pathology.

10. Known history of allergies to dentifrice contents.

Systemically healthy subjects of both genders, between the ages of 18 to 65 years, who were well versed with the use of toothbrush and dentifrice for oral hygiene maintenance, were considered for the study.

Informed consent was obtained from the compliant subjects after explaining the rationale and purpose of the study.

Diagnosis of dentinal hypersensitivity was based on patient’s primary complaint and detailed history of the same regarding subjects’ perception of sensitivity to thermal stimuli (hot or cold), sweet or sour foods, drinks and to tooth-brushing. Other causes of dental pain (caries, periodontal pain) were ruled out during clinical examination. Teeth included in the study had no caries restorations.

To assess tooth sensitivity, a cold pack test of sensitive areas was performed using ice application. Sensitivity was measured using a 10cm visual analog scale (VAS) score, with the score of 0cm being a no-pain response, score of 5cm was perceptible discomfort and a score of 10 being extreme pain or discomfort (modification of Hurkissons VAS, 1974). The clinical examination and sensitivity tests were carried out by a single examiner.

The two toothpastes compared were commercially available non-aqueous toothpaste containing 5% calcium sodium phosphosilicate (test) and commercially available toothpaste containing 5% potassium nitrate (positive control). The present study employed a double blinding procedure to eliminate subjective bias. The brand names from the tubes were painted over with a uniform color by a third person.

Patients reporting a grading of 5 or more of at least 2 teeth (buccal/facial aspects of incisors canines and premolars) were included in the study and designated into Group 1, receiving the toothpaste containing 5% calcium sodium phosphosilicate (tube painted red), and Group 2 receiving the toothpaste containing 5% potassium nitrate (tube painted black) .

Scoring of tooth sensitivity was carried out by using controlled air pressure, from a standard dental syringe ambient temperature, directed perpendicularly and at a distance of 1 to 3 mm from the exposed dentin surface of the test teeth which were duly isolated while adjacent teeth were protected with gloved fingers. VAS scores were recorded at baseline.

Phase I periodontal therapy (scaling) was instituted for all the subject and patients were provided with the respective dentifrice.

The subjects were instructed to brush for 5 minutes, twice daily throughout the period of their study and asked to refrain from consuming very hot, cold, sweet or sour food or drinks. Subjects were also directed to refrain from any other dentifrice or mouthrinse during the trial but were allowed to continue their normal oral hygiene practice.

Assessment was performed again at 2, 4 and 8 weeks.

- Statistical analyses:

Mean VAS scores and mean± S.D. were calculated from individual scores from all subjects in a treatment group. Student paired t-test was used to find out the difference between baseline, 2 weeks, 4 weeks and 8 weeks scores of each group. Mean scores were compared among groups at baseline, 2 weeks, 4 weeks and 8 weeks using One Way Analysis of Variance (ANOVA) to find out the difference between the test and control group with baseline scores. The data of test and control group were compared between 2 weeks, 4 weeks and 8 weeks using Analysis of Co-Variance (ANCOVA) with baseline as covariate. The percentage reduction from baseline- 2 weeks, baseline- 4 weeks and baseline to 8 weeks was compared between the two groups using Students unpaired t-test.

## Results

- Baseline demographics.

A total number of 20 subjects were followed up for a period of 8 weeks. An ANOVA of baseline sensitivity indicated no significant difference effects for the groups for air evaporative stimulus. Since the baselines cores for both groups were similar and did not show any significant differences, these scores were used as a covariate for the ANCOVA.

Significant Improvement Compared to Baseline.

Paired t-tests for each group were carried-out comparing sensitivity at time points two, four and eight weeks to baseline. The trend was increasing reductions in dentin hypersensitivity over time for both test and the positive control group over time. The percentage reduction in sensitivity scores was 40.34%, 57.98% and 75.63% at 2, 4 and 8 weeks respectively for the test group and 24.79%, 47.86% and 64.96%. at 2, 4 and 8 weeks respectively for the positive control group ( Table 1).

**Table 1:**
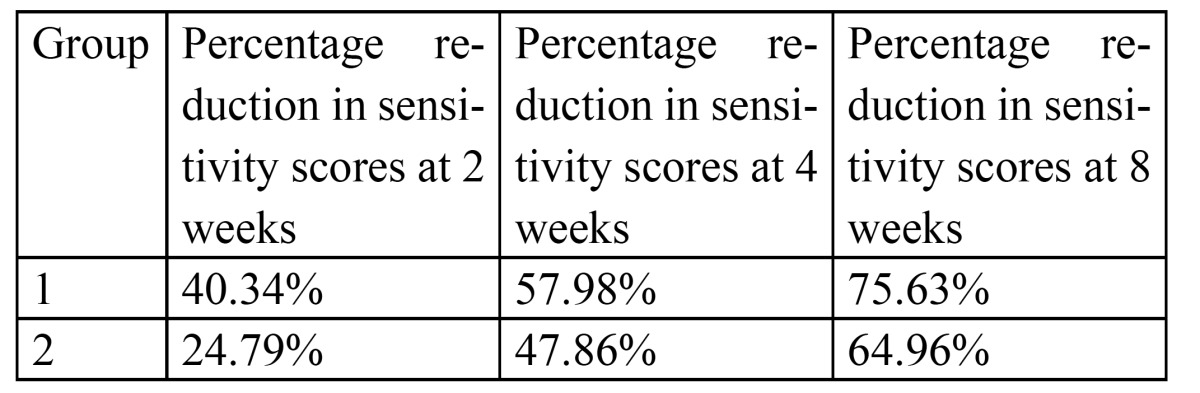
Percentage Reduction in Sensitivity Scores.

- Comparison between groups:

Using an ANCOVA, with data from two weeks, four and eight weeks as dependent variables and baseline values as covariates, the sensitivity scores demonstrated a significant difference among groups at a period of two weeks (p= 0.0207). However there was no significant difference between groups with respect to reduction of scores at 4 and 8 weeks (p values 0.1146 and 0.1152 respectively) ( Table 2).

**Table 2:**
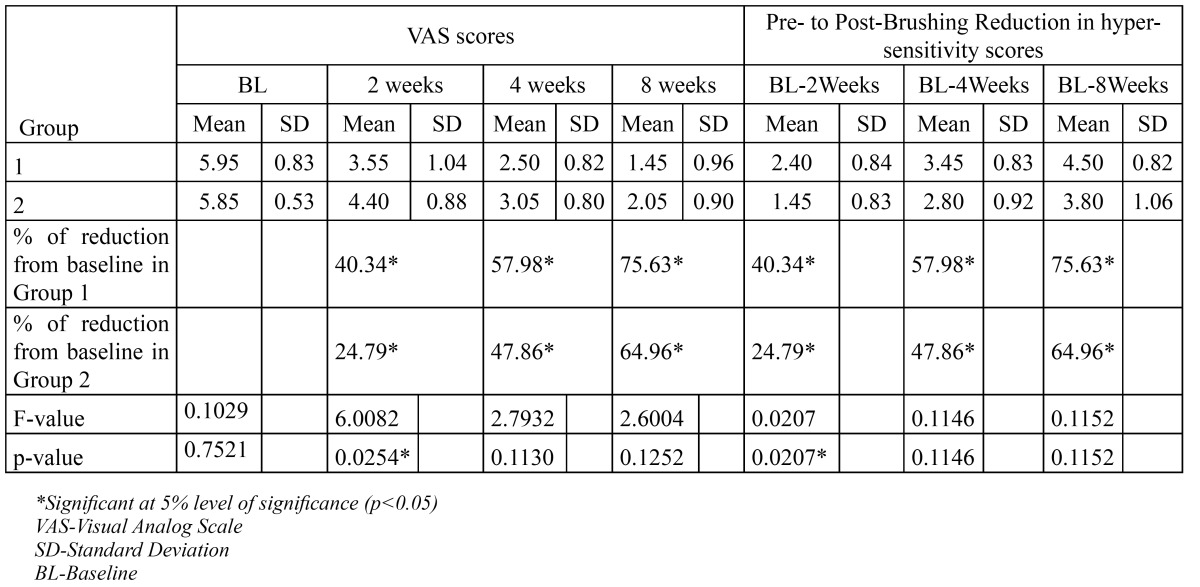
Summary of the pre- and post-brushing hypersensitivity scores for subjects who completed the clinical study.

## Discussion

The objective of this study was to evaluate the temporal efficacy and safety of a new commercially available desensitizing dentifrice formulation containing calcium sodium phosphosilicate toothpaste (Vantej®) and com-pare it to a commercially available potassium nitrate containing toothpaste (Sensodent K®).

Air evaporative stimulus was used to evaluate sensitivity as it can be easily controlled over isolated teeth and simulates the physiologic stimuli. Study duration for this trial was 8 weeks as is recommended by Holland et al. (14).

A review of literature by Gendreau et al. (15), based on randomized controlled clinical trials, support the use of NovaMin in toothpaste formulations in providing relief of pain from dentin hypersensitivity.

The results of the present study demonstrate reduction in symptoms for all treatment groups from baseline to 2, 4 and 8 weeks for measures of sensitivity. There was a remarkable pattern toward reduction of DH with time for all the variables during the 8 weeks of active phase of the study independent of treatment groups. This is in agreement with the studies by Salian et al. (10), Pradeep and Sharma(12), Sharma et al.(16), West et al. (17) and by Litkowski and Greenspan (18).

The calcium sodium phosphosilicate group showed a higher degree of effectiveness at reducing hypersensitivity, than commercially available potassium nitrate for sensitivity, to air evaporative stimulus at 2 weeks. This is in accordance with the results of previous studies which showed that calcium sodium phosphosilicate is more effective compared to potassium nitrate in reducing sensitivity scores as measured using the visual analogue scale (10,12,16). Calcium sodium phosphosilicate toothpaste thus may show greater benefit at an early stage as compared to potassium nitrate (16) which is also advocated in the pilot study by Narongdej et al. (19).

A comparative study by Parkinson and Willson (20) concluded that calcium sodium phosphosilicate imparts significant level of dentinal occlusion with durable occlusive deposits following four days of twice daily brushing in vitro.

However in contrast to the above mentioned studies, there was no significant difference in scores of patients using the calcium sodium phosphosilicate toothpaste as compared to potassium nitrate at 4 weeks and 8 weeks.

This could possibly be due to the fact that both the active agents have been supplied using dentifrice as a delivery vehicle and the excipient (non-active) agents in the dentifrice may serve to occlude dentinal tubules over time, though a previous study has failed to show tubule occlusion with potassium nitrate containing dentifrice (10). Also this effect could be related to a natural decrease in dentin hypersensitivity overtime, or because of patient perception of a decrease in symptoms by virtue of participation in a clinical trial, or may be due to placebo products actually providing some degree of relief from dentin hypersensitivity.

The 5% potassium nitrate toothpaste was used as a positive control in our study because it has proved to be clinically efficient in the treatment of DH (5-8). In our study, the potassium nitrate group showed significant percentage reduction in sensitivity scores but reduction compared to the calcium sodium phosphosilicate group was less at 2 weeks. However, it was found to be as effective as calcium sodium phosphosilicate after 4 and 8 weeks.

The results of the present study may have to be extrapolated with caution given the small sample size and lack of accounting for the placebo effect and the Hawthorne effect. Because calcium sodium phosphosilicate showed greater reduction in sensitivity compared to potassium nitrate at an earlier stage, the direct implication of the present investigation would be faster relief which is of great clinical importance, given the acute nature of dentinal hypersensitivity. Based on the findings of the present study, long term effects of calcium sodium phosphosilicate seem to be less promising than previously claimed.
